# An investigation into mistreatment of women during labour and childbirth in maternity care facilities in Uttar Pradesh, India: a mixed methods study

**DOI:** 10.1186/s12978-019-0668-y

**Published:** 2019-01-23

**Authors:** Gaurav Sharma, Loveday Penn-Kekana, Kaveri Halder, Véronique Filippi

**Affiliations:** 1 Department of Infectious Disease Epidemiology, London School of Hygiene & Tropical Medicine, London, WC 1E 7HT UK; 2 Deputy Manager-Research, Sambodhi Research and Communications, O-2, Lajpat Nagar-II, New Delhi, 110024 India

**Keywords:** Maternal, Newborn, Quality, India, Mistreatment, Disrespect, Abuse, Labour, Childbirth

## Abstract

**Objectives:**

To investigate the nature and context of mistreatment during labour and childbirth at public and private sector maternity facilities in Uttar Pradesh, India.

**Methods:**

This study analyses mixed-methods data obtained through systematic clinical observations and open-ended comments recorded by the observers to describe care provision for 275 mothers and their newborns at 26 hospitals in three districts of Uttar Pradesh from 26 May to 8 July 2015. We conducted a bivariate descriptive analysis of the quantitative data and used a thematic approach to analyse qualitative data.

**Findings:**

All women in the study encountered at least one indicator of mistreatment. There was a high prevalence of not offering birthing position choice (92%) and routine manual exploration of the uterus (80%) in facilities in both sectors. Private sector facilities performed worse than the public sector for not allowing birth companions (*p* = 0.02) and for perineal shaving (*p* = < 0.001), whereas the public sector performed worse for not ensuring adequate privacy (*p* = < 0.001), not informing women prior to a vaginal examination (*p* = 0.01) and for physical violence (*p* = 0.04). Prepared comments by observers provide further contextual insights into the quantitative data, and additional themes of mistreatment, such as deficiencies in infection prevention, lack of analgesia for episiotomy, informal payments and poor hygiene standards at maternity facilities were identified.

**Conclusions:**

Mistreatment of women frequently occurs in both private and public sector facilities. This paper contributes to the literature on mistreatment of women during labour and childbirth at maternity facilities in India by articulating new constructs of overtreatment and under-treatment. There are five key implications of this study. First, a systematic and context-specific effort to measure mistreatment in public and private sector facilities in high burden states in India is required. Second, a training initiative to orient all maternity care personnel to the principles of respectful maternity care would be useful. Third, innovative mechanisms to improve accountability towards respectful maternity care are required. Fourth, participatory community and health system interventions to support respectful maternity care would be useful. Lastly, we note that there needs to be a long-term, sustained investment in health systems so that supportive and enabling work-environments are available to front- line health workers.

## Plain English summary

This study investigated mistreatment during labour and childbirth in public and private sector hospitals in Uttar Pradesh, India.

Two hundred and seventy-five detailed observations of care provided during labour and delivery were conducted by clinical observers using mixed quantitative and qualitative research methods.

This study found that quality of care at the time of birth is generally poor at both public and private sector hospitals in Uttar Pradesh, India. Many inappropriate care practices are routinely utilised while providing maternity care services, which have largely been neglected in policies and programmes so far. There are complex reasons for mistreatment of women at maternity facilities. These include factors associated with policy, infrastructure and resources, ethics, culture, knowledge, skills and attitudes of maternity care providers, and standards at maternity facilities.

Addressing these issues will require long-term investments and focussed action for improvement. As facility-based births and the use of skilled birth attendants continue to rise, a focus on quality and woman-centred maternity care provision is needed to make further improvements.

## Background

The number of maternal deaths remains large in India with 45,000 estimated deaths in 2013 [1]. Since 2006, the Government of India has promoted skilled attendance at birth and rapidly expanded the Janani Suraksha Yojana (JSY) programme that now benefits approximately 40% of India’s birth cohort [2]. The JSY is a cash transfer programme that provides a monetary incentives to women attending institutions for birth [3]. Since 2013, JSY guidelines have been revised and conditionalities associated with parity and minimum age of the mother for institutional deliveries in high and low performing states and union territories have been removed.

However, recent evidence from JSY has been cautionary and highlights the need to improve Quality of Care (QoC), concomitantly, with efforts to increase utilisation of institutional births [4]. Ensuring high QoC at the time of birth encompasses the application of evidence- based obstetric and neonatal care and efforts to ensure positive birth experiences for pregnant woman [5]. Respect, dignity and emotional support, although, integral to ensuring positive birth experiences have been overlooked in research, policy, programmes and practice [6, 7].

There is now increasing research evidence on mistreatment of women during labour and childbirth from both high [8–12] and lower income settings [13–15]. Mistreatment has been previously described as disrespect and abuse [16], obstetric violence [17] and dehumanised care [18]. However, conceptualising what constitutes mistreatment, and therefore, how to measure mistreatment are both complex. A comprehensive definition of mistreatment needs to capture the health, human rights and socio-cultural dimensions of mistreatment, while, measurement efforts need to capture what, where, how and why mistreatment occurs [19]. Freedman et al. have highlighted that measurement efforts should also be able to capture whether mistreatment was intentional or not, and the role of local societal norms (for example- women’s status, patient-provider dynamics) that influences women’s perceptions of mistreatment in different contexts [19].

Given these challenges, a recent WHO systematic review tried to establish the evidence-base for mistreatment globally [11]. They found that most studies use different operational definitions and measurement approaches [11]. Amongst the quantitative studies, only three studies reported a prevalence of mistreatment at maternity facilities, which varied from 15 to 98% [11]. This review also proposed a typology of items considered mistreatment and identified the following: physical, verbal or sexual abuse, stigma and discrimination, lack of informed consent, breaches of confidentiality, neglect and abandonment, refusal to provide pain relief, lack of supportive care, detainment in facilities, bribery and extortion [11].

However, a phenomenon often overlooked in the disrespect and abuse discourse relates to the overuse of inappropriate or unnecessary interventions for care at normal birth. There are examples of health workers in both high and low-income settings underusing simple, inexpensive interventions (for example, birth companionship or counselling on breastfeeding) and overusing ineffective interventions that are more technical, lucrative or convenient despite potential for harm (for example: labour augmentation without indications or caesarean sections) [20–23].

As the 2016 Lancet maternal health series noted, there are two extremes of maternal health care provision in a growing number of LMICs [24]. The first extreme is associated with over-treatment or the routine over-medicalisation of normal labour and births, which they referred as “Too Much Too Soon”. The second extreme is under-treatment or underuse of evidence-based practices signified by the terminology “Too Little, Too Late” which is the underlying cause of high maternal mortality and considerable morbidity [24]. Both over-medicalisation such as increased use of unnecessary procedures like episiotomies without indications or under-treatment such as absent hygienic standards at maternity facilities are also against the rights of child bearing women [25].

For this study, we operationalised mistreatment as those related to 1. disrespect and abuse (no privacy, no birthing position choice, not informing women prior to a vaginal examination, not allowing birth companions, not explaining reasons for augmentation of labour, restricting food and water and informal payments) 2. Overtreatment (routine use of enema, routine use of perineal shaving, application of extreme fundal pressure, routine uterine lavage, routine manual exploration of the uterus and routine episiotomy) and lastly, 3. Under-treatment (deficiencies in infection prevention by individual health workers, deficiencies in hospital environmental hygiene and use of unqualified attendants). Research and programme efforts to improve QoC at the time of birth have largely neglected to examine and address mistreatment in such a comprehensive manner. Further, it is also possible for both under treatment and overtreatment to occur within the same patient and within the same facility [21] which makes interpreting data difficult but this should be considered by researchers working to improve QoC.

Uttar Pradesh (UP) is India’s most populous and deprived state [26]. In related work, we previously described overall poor quality of care at the time of birth [27] but did not specifically examine mistreatment of women at maternity facilities. There are limited number of studies that have described patterns and the context of such care at maternity facilities especially in the private sector which has an estimated 18% of the market share for maternity care in UP [26]. This information is essential for understanding the context of care provision and in developing effective interventions, policy and advocacy approaches for improvement of QoC at the time of birth. Available research evidence indicates that women with previously negative pregnancy outcomes tend to choose private sector [28]. Higher socio-economic status and accessibility are associated with increased private sector use [28]. Scheduled caste and tribe status are negatively associated with use of private facilities [29]. The private sector is thought to be more expensive than the public sector and there is a general perception amongst Indian women that the private sector provides better amenities and a higher standard of care [29].

Qualitative studies from public sector facilities in India have described many challenges to ensuring high QoC during childbirth such as overcrowding of labour rooms, chaotic work environments, poor coordination between health workers, limited skills and competence of health workers in performing routine care procedures [30–32]. They have also described situations where labouring women have been left unsupported, were shouted at or slapped, not given information about what procedures were being done and why they were receiving it [30, 33].

In this paper, we report on the nature and context of mistreatment recorded during 275 clinical observations of labour and childbirth in 26 maternity facilities in Uttar Pradesh. This rich observational data helps us in describing the context of care-provision in a low- resource setting including what, how and why mistreatment of women during labour and childbirth occurs at maternity facilities.

## Methods

### Study setting

The study was conducted in the districts of Kannauj, Kanpur Nagar and Kanpur Dehat of Uttar Pradesh in the context of a large evaluation of the Matrika social franchise programme by the LSHTM [34]. In 2012–2013, the maternal mortality across Uttar Pradesh was 240 per 100,000 live births [26]. At this time, the neonatal mortality rate were 55 per 1000 live births in Kannauj, 41 in Kanpur Nagar and 24 in Kanpur Dehat [26]. Despite government schemes to improve rates of institutional births in public sector facilities, approximately 39% of deliveries in UP (43% in Kannauj, 46% in Kanpur Dehat and 34% in Kanpur Nagar) occurred at public sector facilities in 2012–2013 [26]. The private sector delivery share was estimated to be 18% in UP (15% in Kannauj, 34% in Kanpur Nagar, and 10% in Kanpur Dehat) during that time [26]. The National Rural Health Mission has also appointed community health workers known as Accredited Social Health Activists (ASHAs) in every Indian village [35]. Motivating pregnant women, accompanying them to institutions for childbirth and arranging suitable transportation to hospitals at the start of labour also falls under the responsibilities of ASHAs who are paid a small monetary incentive (INR 600-equivalent £7) for these tasks.

### Sampling

Our sampling frame included all high-volume public sector facilities (> 200 monthly deliveries based on HMIS data [36]) and established private sector facilities providing round-the-clock basic emergency obstetric care identified by Sambodhi Research and Communications (Lucknow, Uttar Pradesh) that has extensive experience of working in health research in the study districts. After mapping of facilities, we selected six public sector facilities per district by conducting a random selection of four community health centres, one medical college and one district hospital and we invited all identified private sector facilities to participate. Since Kanpur Dehat did not have a medical college, we selected an additional district hospital. Amongst the selected facilities, all public sector facilities agreed to participate while 17 private facilities (out of 30) agreed to participate. At nine of the private facilities that agreed to participate, there were no deliveries while observers were present. Therefore, the observational data that we analysed came from 18 public facilities and 8 private sector facilities. Further details on the sampling methods are described elsewhere [27]. The overall study flow diagram is also available in appendix 1.

### Study participants

Study participants included pregnant women with spontaneous, uncomplicated labour (defined as women with low-risk pregnancy, of gestational age between 37 and 42 weeks and singleton vertex presentation, admitted to facilities who consented to participate in the study) and their newborns.

### Data collection

We collected quantitative data from a structured clinical observation tool and qualitative data from open-ended comments recorded by observers. We developed a QoC assessment tool based on a critical assessment of previously used clinical observation tools [37, 38] and WHO guidelines for care during pregnancy and childbirth [39]. This tool captured information on whether maternity care providers correctly performed recommended interventions during the first, second and third stage of labour including the use of practices considered harmful or captured by the mistreatment terminology.

We conceptualised mistreatment of women during labour and childbirth as disrespect and abuse, overtreatment and under treatment during the time of birth as described previously. Specifically, our questionnaire captured information on ensuring adequate privacy, explaining the process of labour, restricting food and fluids, informing women prior to vaginal examination and prior to labour augmentation, performing an enema, perineal shaving, not allowing a birth companion, not offering choice of birthing position, routine episiotomy, physical abuse (slapping or hitting), verbal abuse (insult, threaten and shout), routine application of fundal pressure, routine uterine lavage and routine manual exploration of the uterus after childbirth.

Questions capturing educational, demographic and socio-economic status were adapted from the National Family Health Survey questionnaire [40]. At the end of every case, clinical observers who were auxiliary nurse midwives and had maternal and child health backgrounds were encouraged to record open-ended comments about the QoC they observed, particularly, anything they felt was important to explain the context and things that were particularly striking to them. Observers had been trained on the concepts of respectful maternity care including disrespect and abuse during field-level trainings [25]. A team of 14 clinical observers working in pairs at each facility observed care round the clock. They visited the admissions, emergency, labour room and postnatal wards to identify pregnant women who were likely to undergo uncomplicated vaginal births and observed care provided from admission to one hour postpartum. Data were collected after obtaining women’s informed written consent between 26th of May to 8th of July 2015.

### Ethics

Ethical approval was obtained from the Public Healthcare Society (PHS) Ethics Review Board in India and the London School of Hygiene and Tropical Medicine in the UK (LSHTM Ethics Ref: 8858). The study also received government clearance from the National Health Mission in Uttar Pradesh.

## Analysis

### Measurement

We collected data on 15 potentially harmful interventions as outlined previously. Each item was coded as 1 if observed and 0 otherwise. An aggregate measure of mistreatment was developed which was the mean of observed items of mistreatment for every woman (Range: 0–15). Potential covariates included women’s age, parity, referral status, caste, socio-economic status, delivery by qualified personnel, admission during work-hours, admission during weekends and public or private sector. For socio-economic status, wealth quintiles were generated using principal component analysis using data on ownership of household assets [41].

### Quantitative analysis

Descriptive analyses were carried out at the level of individual women using STATA 14 (Stata Corp. LP, College Station, United States of America). Since preliminary analysis showed that all women encountered at least one item of mistreatment (Appendix 2), we categorised the sample into two groups based on the median number of items of mistreatment observed, as shown in Table 1. We then conducted a bivariate analysis to examine the relationship between indicators of mistreatment and socio-demographic characteristics. Means, proportions and a total mistreatment score were calculated for all covariates. Chi square tests were used to assess whether there was a significant difference amongst the use of practices considered mistreatment and the relevant co-variates.

**Table 1:** Socio-demographic characteristics of the sample by two overall levels of mistreatment

	Total (*n* = 275) N, (%)	Less than or equal to median number of mistreatment items N, (%)	Greater than median number of mistreatment items N, (%)	*P*^a^ value
1. Women’s age				
a. < 20 years	16 (5.8)	14 (7.5)	2 (2.3)	0.23
b. 20–35 years	247 (89.8)	165 (88.2)	82 (93.2)	
c. 35 years or more	12 (4.4)	8 (4.3)	4 (4.6)	
2. Parity				
a. Primipara	119 (43.3)	76 (40.6)	43 (48.9)	0.32
b. Multipara	145 (52.7)	102 (54.6)	43 (48.9)	
c. Grandmultipara	11 (4.0)	9 (4.8)	2 (2.3)	
3. Referral status				
a. Patient comes directly to this facility	243 (88.4)	164 (87.7)	79 (89.8)	0.62
b. Patient referred from another facility	32 (11.6)	23 (12.3)	9 (10.2)	
4. Caste group^b^				
*a. “Scheduled caste and tribe”*	61 (22.2)	38 (20.3)	23 (26.1)	0.40
*b. “Other backward caste”*	153 (55.6)	109(58.3)	44 (50.0)	
*c. “General caste”*	61 (22.2)	40 (21.4)	21 (23.9)	
5. Socio-economic status				
a. 1st quintile (lowest)	56 (20.4)	41 (21.9)	15 (17.1)	0.56
b. 2nd quintile	54 (19.6)	35 (18.7)	19 (21.6)	
c. 3rd quintile	55 (20.0)	39 (20.9)	16 (18.2)	
d. 4th quintile	55 (20.0)	39 (20.9)	16 (18.2)	
e. 5th quintile (highest)	55 (20.0)	33 (17.7)	22 (25.0)	
6. Delivery by qualified attendants				
a. Qualified attendants ^c^	113 (41.1)	78 (41.7)	35 (39.8)	0.76
b. Unqualified attendants ^d^	162 (58.9)	109 (58.3)	53 (60.2)	
7. Timing of admission				
a. Within work hours (9:00 AM − 17:00 PM)	254 (92.4)	168 (89.8)	86 (97.7)	0.02
b. Out of hours (17:01 PM to 8: 59 am)	21 (7.6)	19 (10.2)	2 (2.3)	
8. Admission day				
a. Admission during weekdays	211 (76.7)	141 (75.4)	70 (79.6)	0.45
b. Admission during weekends.	64 (23.3)	46 (24.6)	18 (20.5)	
9. Sector				
a. Public	211 (76.7%)	138 (73.8)	73 (82.9)	0.09
b. Private	64 (23.2%)	49 (26.2)	15 (17.1)	

^a^For the comparison of the proportions for less than or equal to median number of items of mistreatment observed and greater than median number of items of mistreatment that were observed

^b^The caste system in India is a system of social stratification that places people in occupational groups. Members of *scheduled* castes are the lowest castes in society and protected by the government through special concessions [61]. For caste, we have used the exact language of the various ethnic categories given in Indian national family health survey questionnaires

^c^Doctors, nurses or nurse-midwives – with at least 5, 4 and 2 years of pre-service training, respectively – who are licensed, regulated and endorsed by the government to provide maternity care at health facilities

^d^Accredited social health activists, cleaners, hospital porters, other community health workers, traditional birth attendants and others who are not legally allowed by the government to provide maternity care at health facilities

### Qualitative analysis

The open-ended comments were transcribed in Hindi and translated to English and analysed using Nvivo 11 software (QSR International). A thematic analysis approach was utilised. Two researchers (GS, LPK) independently reviewed comments line- by- line and then agreed on a set of codes; broadly categorised into those related to the quantitative checklist and codes for other emerging issues. Both researchers then jointly coded all the open-ended comments. In cases where disagreements arose between researchers, further discussion took place until consensus was achieved. Throughout the analysis process, researchers reflected on how their background, training and worldview might influence their interpretation of results and efforts were taken to minimise them. We triangulated the quantitative data with qualitative comments. Comments that summarise common findings across observations are reported.

## Results

We first report on women’s socio-demographic characteristics categorised by two overall mistreatment levels. Next, we present bivariate analysis of the prevalence of specific indicators of mistreatment for which quantitative data are available and examine their relationship with socio-demographic characteristics of the sample. Then we report on our qualitative findings which provide additional information and triangulate these to the quantitative results, where possible, to further explain the nature and the context in which mistreatment occurs.

1. Demographic characteristics

The majority of observations were conducted in the public sector (*n* = 211, 77%) and most women came directly to facilities (88%) (Table 1). Amongst our sample, the majority of participants were between 20 and 35 years of age (90%), multi-parous (53%), came directly to the facility (88%), belonged to the so-called “other backward caste” category (55%) and were from the lowest wealth quintile (20%). Most deliveries were performed by unqualified personnel (59%) during regular work-hours (92%) on weekdays (77%). The only variable significantly different was timing of admission and a greater proportion of mistreatment was observed in cases admitted during work hours compared to observations done beyond regular working hours (*p* = 0.02).

2. Patterns of mistreatment by socio-demographic characteristics

**Fig. 1:**
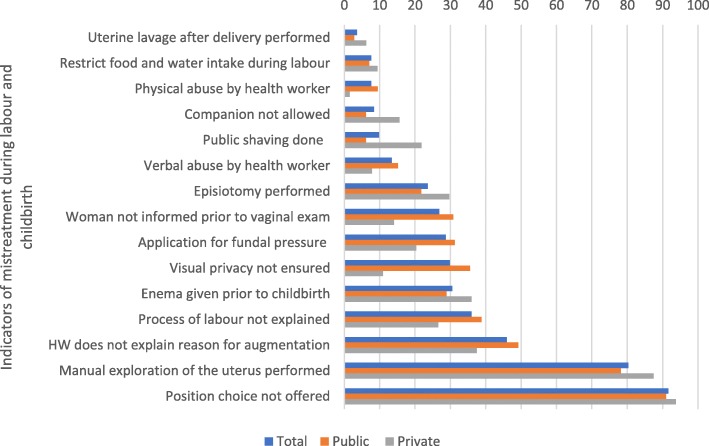
Quantitative results showing the prevalence of indicators of mistreatment in public and private sector maternity facilities

Figure 1 shows that amongst mistreatment practices, birthing position choice not offered to the labouring woman (92%) and manual exploration of the uterus after delivery (80%) were particularly high at facilities in both sectors.

Table two shows that amongst all socio-demographic characteristics, the highest mistreatment scores (mean) for women, were found in women above 35 years of age (5.1); primiparous (5.2);those that were referred from another facility (5.0); amongst women belonging to “scheduled caste and tribes” (5.0), those in the fifth (richest) wealth quintile (5.1), and amongst cases admitted during work-hours (5.0) on weekdays (5.0) in the public sector (4.9). However, the timing of admission (during weekdays or weekends) influenced a greater number of indicators of mistreatment compared to admission during regular work-hours, despite total mistreatment scores being similar across both co-variates. More women admitted during weekdays underwent episiotomies (*p* = 0.04) and enemas (*p* = 0.01) whereas, more women admitted during weekends were not informed prior to vaginal examination (*p* = 0.03) and did not have the process of labour explained to them (*p* = 0.04). We found that more women admitted during regular work-hours delivered without adequate privacy (*p* = 0.01), underwent enemas (*p* = 0.03) and extreme fundal pressure (*p* = 0.01) more frequently. Most women had repeat instances of mistreatment (mean = 4.8 and SD = 1.7).

Table 2 shows that the public sector performed worse than the private sector for not ensuring privacy of the labouring women (p = < 0.001), not informing women prior to a vaginal examination (p = 0.01) and for physical violence (shout, hit or pinch) towards the labouring woman (*p* = 0.04). On the other hand, the private sector performed worse than the public sector for not allowing birth companions to accompany the labouring woman (*p* = 0.02) and for perineal shaving (*p* = < 0.001).

**Table 2:** Bivariate analysis of the significance by socio-demographic factors and the prevalence of observed indicators of mistreatment

	No privacy %	No Position choice %	Woman not informed prior to vaginal exam %	Companion not allowed %	Process of labour not explained %	Reason for augmentation not explained %	Restrict food and water %	Enema %	Public shaving %	Fundal pressure %	Uterine lavage %	Manual uterus exploration %	Episiotomy %	Physical abuse %	Verbal abuse %	Mistreatment score (mean)
Total N reporting mistreatment (N = 275)	82	252	74	23	99	40	21	84	27	79	10	221	65	21	37	Range 1–15
Women’s age																
< 20 years	18.8%	81.3%	25.0%	0.0%	18.8%	12.5%	0.0%	62.5%	6.3%	18.8%	0.0%	68.8%	43.8%	0.0%	0.0%	4.4
20–35 years	30.4%	92.3%	27.1%	8.9%	36.0%	15.0%	8.5%	28.7%	10.5%	28.7%	4.0%	81.4%	23.1%	7.3%	14.2%	4.9
35 years or more	33.3%	91.7%	25.0%	8.3%	58.3%	8.3%	0.0%	25.0%	0.0%	41.7%	0.0%	75.0%	8.3%	25.0%	16.7%	5.1
Pearson Chi square	0.59	0.30	0.97	0.46	0.10	0.79	0.28	0.02	0.43	0.42	0.56	0.42	0.08	0.04	0.26	
Parity																
Primipara	26.1%	91.6%	24.4%	9.2%	31.9%	20.2%	6.7%	36.1%	16.%	34.5%	5.0%	80.7%	45.4%	7.6%	16.0%	5.2
Multipara	33.1%	91.0%	30.3%	8.3%	41.4%	10.3%	7.6%	24.1%	4.8%	25.5%	2.8%	78.6%	7.6%	8.3%	11.7%	4.7
Grandmultipara	27.3%	100.0%	9.1%	0.0%	9.1%	9.1%	18.%	54.5%	0.0%	9.1%	0.0%	100.0%	0.0%	0.0%	9.1%	4.3
Pearson Chi square	0.45	0.59	0.22	0.57	0.05	0.07	0.39	0.02	0.003	0.10	0.50	0.23	< 0.001	0.61	0.55	
Referral status																
Patient comes directly to this facility	29.6%	91.8%	27.2%	7.4%	36.6%	13.2%	7.4%	30.0%	9.9%	30.0%	2.9%	79.8%	21.8%	7.4%	12.3%	4.9
Patient referred from another facility	31.3%	90.6%	25.0%	15.6%	31.3%	25.0%	9.4%	34.4%	9.4%	18.8%	9.4%	84.4%	37.5%	9.4%	21.9%	5.0
Pearson Chi square	0.85	0.83	0.80	0.11	0.55	0.07	0.69	0.62	0.93	0.19	0.07	0.54	0.05	0.69	0.14	
Caste																
*“Scheduled caste and tribe”*	32.8%	93.4%	36.1%	8.2%	39.3%	13.1%	9.8%	27.9%	6.6%	34.4%	1.6%	78.7%	19.7%	11.5%	13.1%	5.0
*“Other backward caste”*	28.1%	92.2%	24.2%	6.5%	35.3%	13.1%	8.5%	30.1%	10.5%	24.2%	3.9%	82.4%	20.3%	6.5%	15.0%	4.8
*“General caste”*	31.1%	88.5%	24.6%	13.1%	34.4%	19.7%	3.3%	34.4%	11.5%	34.4%	4.9%	77.0%	36.1%	6.6%	9.8%	4.9
Pearson Chi square	0.77	0.58	0.19	0.11	0.82	0.44	0.33	0.72	0.61	0.18	0.60	0.63	0.04	0.44	0.60	
Socio-economic status																
1st quintile (lowest)	41.1%	89.3%	42.9%	7.1%	46.4%	17.9%	5.4%	25.0%	8.9%	30.4%	0.0%	83.9%	10.7%	3.6%	12.5%	4.9
2nd quintile	27.8%	90.7%	37.0%	3.7%	33.3%	11.1%	7.4%	29.6%	3.7%	27.8%	5.6%	74.1%	16.7%	14.8%	20.4%	4.8
3rd quintile	23.6%	96.4%	18.2%	5.5%	43.6%	12.7%	12.7%	38.2%	5.5%	20.0%	9.1%	74.5%	25.5%	3.6%	7.3%	4.7
4th quintile	32.7%	92.7%	21.8%	5.5%	32.7%	12.7%	7.3%	20.0%	5.5%	30.9%	3.6%	83.6%	21.8%	10.9%	16.4%	4.8
5th quintile (highest)	23.6%	89.1%	14.5%	20.0%	23.6%	18.2%	5.5%	40.0%	25.5%	34.5%	0.0%	85.5%	43.6%	5.5%	10.9%	5.1
Pearson Chi square	0.22	0.62	0.002	0.01	0.09	0.76	0.59	0.11	0.001	0.53	0.05	0.37	0.001	0.10	0.31	
Delivery by qualified attendants *																
Unqualified attendants	30.2%	93.2%	32.7%	4.9%	36.4%	15.4%	9.3%	28.4%	6.2%	29.0%	1.9%	78.4%	17.3%	9.9%	16.0%	4.8
Qualified attendants	29.2%	89.4%	18.6%	13.3%	35.4%	13.3%	5.3%	33.6%	15.0%	28.3%	6.2%	83.2%	32.7%	4.4%	9.7%	4.9
Pearson Chi square	0.85	0.26	0.01	0.01	0.86	0.62	0.23	0.35	0.02	0.90	0.06	0.33	0.003	0.09	0.13	
Admission during work hours^#^																
Within work hours	31.9%	90.9%	28.0%	9.1%	36.2%	15.0%	7.1%	32.3%	10.6%	30.7%	3.9%	80.7%	24.8%	7.9%	13.8%	5.0
Out of hours	4.8%	100.0%	14.3%	0.0%	33.3%	9.5%	14.3%	9.5%	0.0%	4.8%	0.0%	76.2%	9.5%	4.8%	9.5%	3.7
Pearson Chi square	0.01	0.15	0.18	0.15	0.79	0.50	0.23	0.03	0.12	0.01	0.35	0.62	0.11	0.61	0.58	
Admission during weekends?																
Admission during weekdays	30.8%	90.0%	23.7%	10.0%	32.7%	14.2%	7.1%	34.6%	11.%	29.4%	4.7%	82.0%	26.5%	8.5%	14.7%	5.0
Admission during weekends.	26.6%	96.9%	37.5%	3.1%	46.9%	15.6%	9.4%	17.2%	4.7%	26.6%	0.0%	75.0%	14.1%	4.7%	9.4%	4.6
Pearson Chi square	0.52	0.08	0.03	0.08	0.04	0.78	0.55	0.01	0.12	0.66	0.08	0.22	0.04	0.31	0.28	
Sector																
Public sector	35.5%	91.0%	30.8%	6.2%	38.9%	14.7%	7.1%	28.9%	6.2%	31.3%	2.8%	78.2%	21.8%	9.5%	15.2%	4.9
Private sector	10.9%	93.8%	14.1%	15.6%	26.6%	14.1%	9.4%	35.9%	21.%	20.3%	6.3%	87.5%	29.7%	1.6%	7.8%	4.7
Pearson Chi square	< 0.01	0.49	0.01	0.02	0.07	0.90	0.55	0.29	< 0.01	0.09	0.20	0.10	0.19	0.04	0.13	

Our data shows that the highest mistreatment scores were amongst women that came to district hospitals (6.1) where they experienced higher rates of no privacy (*p* = < 0.001), not being informed prior to vaginal examination (0.001), using unsterile gloves to conduct vaginal examinations (*p* = 0.031), application of fundal pressure (< 0.001) and episiotomies (*p* = < 0.001).

3. Specific patterns of mistreatment that occur at maternity facilities

The section below summarises qualitative information obtained from observers’ open-ended comments on mistreatment. It provides contextual insights into the quantitative data presented earlier, as well as additional information on categories and themes of mistreatment such as deficiencies in infection prevention, lack of analgesia for episiotomy, informal payments and poor health facility environmental hygiene which were not captured by the quantitative checklist (Table 3).

1. **Overtreatment by health workers**

- Fundal Pressure:

**Table 3:** Themes and their composition- clinical observations of labour and childbirth at maternity facilities

Categories	Themes	Composition
1. Over-treatment	a) Extreme fundal pressure	Occurs frequently and help often sought from others present
	b) Routine episiotomy	Occurs frequently and often conducted without any analgesia.
2. Under-treatment	c) Deficiencies in Infection prevention by individual health workers	Using dirty clothes to clean the perineal and vaginal areas, unhygienic care procedures, conducting unnecessary procedures without proper infection prevention measures and using unsterile gloves and equipment.
	d) Unqualified birth attendants	Chronic staff shortages mean that unqualified health workers are often involved providing maternity care services.
	e) Health facility environmental hygiene	Limited adherence to infection management protocols, no facilities for hand washing, no use of antiseptics, non-availability of protective gear, inadequate sterilisation of equipment’s, aprons or facemasks, no waste disposal systems and stray animals such as dogs and cows in premises.
3. Disrespect and abuse	f) Physical violence and verbal abuse	Health workers are often anxious and sometimes use physical violence and verbal abuse. Physical abuse ranged from slapping the pregnant woman, to hitting and pinching her thighs or restraining forcefully. Verbal abuse ranged from talking down to the pregnant woman, using foul language and threatening women with caesarean sections, if they did not stop shouting or crying.
	g) Informal payments	Frequent in public sector maternity facilities. These range from Rupees 200–2000, equivalent £2.4 to £24

Our quantitative results (Fig. 1) show that the prevalence of fundal pressure was 29%; similar across both sectors (*p* = 0.09) but done more frequently during regular work- hours (*p* = 0.01) compared to outside regular work hours. The descriptions of fundal pressure recorded by observers in open-ended comments ranged from application of light pressure to extreme pressure on the upper abdomen directed downwards to the birth canal. In a few instances, observers noted that maternity care personnel climbed on top of the bed and use both hands to push down forcefully on the abdomen. Often physical violence was also used while performing fundal pressure. Although, fundal pressure was mostly done by personnel attending to the delivery, help was also sought from others present in the labour room such as mother-in laws and ayahs. The circumstances leading to the decision to apply extreme fundal pressure included to expedite the delivery process, when the woman could not tolerate labour pains or could not bear down or push properly. The quote below illustrates an example of how fundal pressure was described in the field notes.

*The physical state of the labour room of the district hospital is poor. They give fundal pressure on the abdomen the way people use pumps for filling air in cycle tyres. They were pressing their abdomen with their elbows during delivery and also slapped the lady badly.* (Clinical observation of 35-year-old, primi at district hospital.)

b) **Episiotomy:**

Quantitative results indicate that episiotomy was done in 24% of cases and that the prevalence was similar across both sectors (*p* = 0.19). However, amongst cases where episiotomy was given, no analgesia was given in 25% of cases, similar across both sectors (*p* = 0.09). Comments recorded by observers corroborate that analgesics were often not given during episiotomies despite women crying and shouting in pain. Anecdotal evidence collected during fieldwork suggests that health workers seem to believe that women do not require analgesia during episiotomy as they are already in so much pain and will not feel any additional pain. The quote below illustrates an example of episiotomy recorded in field notes.

*“Episiotomy was conducted without analgesia because of which the patient was constantly shouting. The nurse consoled her saying it was only a few stitches, but no analgesia was given and instead the nurse scolded her before giving her stitches”* (Clinical observation at a district hospital in a 34-year multigravida woman.)

2. **Under treatment:**

c) Deficiencies in infection prevention:

Deficiencies in infection prevention by individual health workers was also an important theme in the observers’ comments. These deficiencies by individual health workers ranged from using dirty clothes to clean the perineal and vaginal areas, pouring oil over the vagina/ perineum, conducting unnecessary manual exploration of uterus, and using unsterile gloves and equipment. Although quantitative data is not available for all of these practices, available quantitative results suggest deficiencies in infection prevention measures while conducting unnecessary procedures. For example, there was a high prevalence (80%) of manual exploration of the uterus which was similar in both sectors (*p* = 0.10). Enemas were also observed in 30% of cases risking possible faecal contamination. It is encouraging to note that most health workers used sterile gloves; use of unsterile gloves to conduct vaginal examinations was low and happened in just 2.2% of all cases, all in the public sector (3%). Uterine lavage after delivery was also infrequent in both public (3%) and private sectors (6.3%) cases. Observers’ comments also indicate that in some facilities, instruments were sterilised once a day and often just dipped in warm water and chlorhexidine solution and reused multiple times. Vaginal examinations were observed to be conducted multiple times by different health workers. In a few instances, observers’ comments mention that used syringes were left discarded on the floor, which is a potential hazard for needle-stick injuries.

The quotes below illustrates some examples of deficiencies in infection prevention by individual health workers:

*“While suturing the episiotomy, ayah accepted a phone call, also touched the bed with her gloved hands and then continued with the suturing. Manual exploration of the placenta was also done to check whether anything was left inside”* (Clinical observation in a community health centre of a 28-year-old multiparous woman).

*“Here, gloves are taken out from the powder. I don’t know if they use autoclaves. They did not inform me. They just wash instruments with water only. Mostly they dip instruments in warm water, but the blood stains are still there. Cheatle forceps are available but they do not keep it properly.”* (Clinical observation at a district hospital in a 30-year-old grand multiparous woman).

d) **Health facility environmental hygiene:**

The wider facility environment and hospital infection prevention and control measures were also noted as a serious concern in many of the observers’ comments. This theme captures issues beyond the control of the individual health workers such as those at the institutional level, and has been conceptualised as under-treatment, which constitutes mistreatment of women, since it is unethical to allow women to deliver in such unhygienic conditions. Observers’ comments frequently describe limited adherence to infection management protocols at facilities, no facilities for hand washing, no use of antiseptics, non-availability of protective gear, inadequate sterilisation of equipment’s, aprons or facemasks. Systems for segregation of wastes (used injection vials, sharp instruments or wastes such as placenta, other fluids) such as colour-coded bins were non-functional. A frequent finding was that stray animals such as dogs and cows roamed throughout the facility compound and often took shelter in the wards or labour rooms. Clean towels and sterile pads were frequently not available at hospitals; instead, women’s old clothes such as old saris were used to wipe the woman and newborn after childbirth. Suction machines and radiant warmers, even when available, were often found to be unused and dirty. Beds sheets were not changed regularly and multiple women were observed giving birth in the same bed. The quotes below illustrate some examples of comments recorded under this theme.

*“Instruments here are neither washed properly nor placed in the autoclave. They clean it with water and use them again. Doctor, nurse, ayah - none of them take care of anything. There is no water available in the bathroom. No one cleans the bed after delivery for next patient. Another woman was asked to lay over the same bed where there was blood from the previous delivery.”* (Clinical observation at a Community health centre of a 25-year-old multiparous woman).

*“The hospital is private but it doesn’t look like one other private hospitals. Repeated deliveries are conducted without even cleaning the bed properly. In the labour room, the staff chew and spit tobacco and there are stains everywhere. There is a large focus light in the labour room which is covered with dust. There are mice in the labour room. They never use the autoclave machine although it is available.”* (Clinical observation at a private hospital of 27-year-old multiparous woman.)

e) **Unqualified birth attendants:**

Quantitative data indicate that 59% of all births were attended by unqualified personnel, more frequently in the public (64%) than the private (41%) sector (*p* = 0.001). We conceptualised the use of unqualified personnel as under-treatment because it is unethical for women to received care from unqualified personnel at institutions. Our findings indicate that given the chronic staff shortages, the role of unqualified personnel seems important and established in the provision of care during labour and childbirth. The sweeper, traditional birth attendant (dai) and the ayah (helper) tend to be involved in supporting work in the labour room such as bringing instruments or delivery trays when the delivery is imminent. They are often also involved in conducting the deliveries when the doctors and nurses are not available or do not attend normal deliveries. The quotes below highlight some examples from field notes.

*“After examining the pregnant woman, the nurse asked if any dai had checked her as well. Dais are routinely involved in providing care at this facility. I did not observe any doctors during my shift”* (Clinical observation at a community health centre of a 25-year-old multiparous woman).

*“Nurses of this private hospital are not trained. They are studying now and are working based on some experience.”* (Clinical observation in a private hospital of a 26-year-old primiparous woman).

3. **Disrespect and abuse**

f) Physical violence and verbal abuse

Physical violence and verbal abuse were a common theme in observer’s comments. From the quantitative data, the prevalence of physical abuse was 7.6%; and more frequent in the public sector than the private sector (*p* = 0.04) and greater amongst women above 35 years of age (p = 0.04). Although, verbal abuse was also more prevalent in the public sector (15%) than in the private sector (8%), this was not statistically significant (*p* = 0.13). The descriptions of physical violence in the open-ended comments ranged from slapping the pregnant woman to hitting and pinching her thighs while she was bearing down. Slapping often occurred while fundal pressure was being applied. Verbal abuse ranged from talking down to the pregnant woman, using foul language and threatening women with caesarean sections, if they did not stop shouting or crying. In most instances, field-researchers noted that staff appeared anxious at the time of the birth and often used physical violence (such as slapping, forcing woman to bear down or restraining the woman) during the birthing process. There were no instances recorded in the field notes where pregnant woman or their companions stood up to mistreatment or abuse by health workers. The quotes below illustrate physical violence, verbal abuse and mistreatment of pregnant woman encountered during clinical observations.

*“The nurse said, when you are with your husbands, you don’t shout but you are shouting now. You will come again with another baby soon!”* (Clinical observation at a district hospital of a 27-year-old multiparous woman.)

*“The nurse was badly scolding the pregnant woman. The women appeared restless and was screaming and shouting. The nurse threatened her and said that if she continues to scream, she would operate on her.”* (Clinical observation at a district hospital in a 25-year-old primiparous woman)

g) **Informal payments:**

The practice of maternity care personnel asking for informal payments at public sector facilities was the most common theme identified from the observers’ comments and is a form of disrespect and abuse. However, quantitative data capturing this phenomenon were not captured during clinical observations. Observers’ comments indicate that in most instances, maternity care personnel demanded money from families for doing activities that are a part of their job description such as drying and wrapping the newborn, weighing the newborn, cleaning blood spills on the delivery bed or labour room floor and cleaning up. Often in public-sector hospitals, maternity care personnel demanded money from clients and their families to cover their costs, as they were contractual staff, allegedly, without a regular monthly income source. In some instances, informal payments were also given to health workers as gratuity payments given to express happiness at the birth of newborn.

Field notes also indicate that there is an understanding between the maternity care personnel and community health worker such as ASHAs who often act as the intermediary between the clients and health workers, facilitating the exchange of such payments. In addition, in most observations, families were asked to purchase drugs and commodities such as gloves, baby towels, medicines, delivery kits from outside, although, in principle these items should be provided free of cost at health facilities under the JSY scheme. There were also a few cases where observers documented that newborns were withheld from families until providers received payments from families. If the providers did not receive money, women were more likely to be mistreated during their hospital stay. The amount of informal payments varied between Rupees 200–2000, equivalent £2.4 to £24. The quotes below illustrate some examples of the practices of informal payments at maternity facilities.

*The junior nurses ask for money in this hospital. They say – “Give me Rs.2000. We have performed the delivery so well. If we had not done that the child would have died inside, you. I will take half of the money and will give the rest to madam.”* (Clinical observation at a district hospital of a 22-year-old multiparous woman).

*“Nurse was fighting for money. She conducted delivery only after receiving money. Family members are asked to bring clothes for cleaning mother and child. Money for gloves is also taken from family members.”* (Clinical observation at a community health centre of a 23-year-old primiparous woman.)

## Discussion

This study explored the nature and context of mistreatment amongst women attending public and private sector maternity facilities in Uttar Pradesh. All women in the study encountered at least one indicator of mistreatment. Our estimates are similar to another cross-sectional study from a teaching hospital in south-eastern Nigeria where 98% of women reported some kind of mistreatment during childbirth [42]. Similarly, another cross-sectional study in Ethiopia also found a high prevalence of mistreatment where 100% of women that went to a teaching hospital and 89.4% that went to peripheral health centres encountered some form of mistreatment [43]. The prevalence of mistreatment reported across studies varies depending on how mistreatment is conceptualised and measured [11]. A recent cross-sectional study from Uttar Pradesh, India reported that 57% of urban slum-resident women reported some form of perceived mistreatment during childbirth [44]. In Tanzania, researchers found 19% perceived mistreatment amongst a sample of women while using hospital-exit interviews and up to 28% mistreatment amongst the same women followed-up at home which they attribute to courtesy bias in the exit interviews [45]. However, unlike in our study, both of these studies measured perceived mistreatment rather than direct observations of labour and childbirth.

We found that total mistreatment scores were higher amongst women attending district hospitals (6.14), women above than 35 years of age (5.1), primiparous (5.2), those that were referred from another facility (5.0), amongst women belonging to the “scheduled caste and tribe” (5.0), those in the fifth (richest) wealth quintile (5.1), and amongst cases admitted during work-hours (5.0) on weekdays (5.0) in the public sector (4.9). The cross-sectional study from urban slums in Uttar Pradesh, mentioned earlier also found that wealthier women, migrant women and women from lower castes reported higher levels of disrespect and abuse [44]. The importance of caste is well documented in India with many studies reporting inferior care and discrimination against women belonging to these so-called scheduled castes [44, 46–48]. Researchers have suggested that since these women are less empowered, health workers are more likely to think that they can get away with mistreatment of these women [44].

We found that not offering woman a choice of birthing position (92%) and manual exploration of the uterus after delivery (80%) were particularly high at facilities in both sectors. There is evidence from a systematic review supporting the benefits of delivering in alternative positions compared to supine positions for normal births such as shorter labour duration, fewer episiotomies and fewer second-degree tears [49]. Manual exploration of the uterus is an important risk factor for puerperal sepsis and shock [50] and should be avoided unless indicated and constitutes overtreatment which is form of mistreatment. Further, it is essential to provide all women with adequate information and obtain an informed consent prior to any invasive clinical procedure such a vaginal examination [50].

We found that the public sector performed worse than the private sector for not ensuring privacy of the labouring women (p = < 0.001), not informing women prior to a vaginal examination (*p* = 0.01) and for physical violence (shout, hit or pinch) towards the labouring woman (*p* = 0.04). There could be many reasons for poor performance of the public sector such as inadequate infrastructure, high-workloads, poor communication skills and normalisation of disrespect and abuse in actual practice. During fieldwork, we noted that public sector facilities were crowded and that maternity care personnel worked in challenging environments often without basic amenities, limited incentives and these environments were not conducive to practice evidence based maternity care.

On the other hand, the private sector was found to perform worse than the public sector for not allowing birth companions to accompany the labouring woman (*p* = 0.02) and for perineal shaving (*p* = < 0.001). This could perhaps be due to existing institutional polices in private hospital labour rooms which do not allow birth companions. A recent Cochrane review found that that continuous support from a chosen family member or a friend increased women’s satisfaction with their childbearing experience [51]. Although, performed with the belief that perineal shaving reduces the risk of infection, a systematic review has found no associated clinical benefits of shaving [52]. Perineal shaving is also recommended-against in the Indian skilled birth attendance training materials [53], which suggests that, perhaps, private sector health workers may not have received these trainings or that quality of such trainings is poor. It is also possible that perineal shaving is done more frequently in the private sector as a way of demonstrating that they provide better value for money.

We also found some interesting associations between women’s socio demographic characteristics and the prevalence of specific indicators of mistreatment. Caste was only associated with episiotomy and women in the so-called “general caste” were found to have greater proportions of routine episiotomies (*p* = 0.04) perhaps because they used public sector facilities more often. Women in the first quintile (poorest) were least likely to be informed prior to a vaginal exam (*p* = 0.002) which suggests discriminatory care based on wealth status [47]. However, women in the highest wealth quintile (richest) were more frequently unaccompanied by a birth companions (*p* = 0.01), had higher rates of perineal shaving (*p* = 0.001) and episiotomy (*p* = 0.001) which could perhaps reflect greater use of the private sector and consequent overtreatment of women that attend private sector facilities.

Mistreatment was found to be higher amongst women > 35 years perhaps because they had higher rates of physical abuse (*p* = 0.04) compared to women in other age groups. Primiparas also received higher mistreatment scores because they had higher rates of episiotomies (*p* < 0.001) and pubic shaving (*p* = 0.003) compared to women with higher parity. Women in the fifth wealth quintile (highest) also received higher mistreatment scores compared to other women because they had higher rates of episiotomies (*p* = 0.001), pubic shaving (p = 0.001) and were not allowed birth companions (*p* = 0.01), probably a reflection of where they went for labour.

Overall mistreatment scores were marginally higher for qualified attendants (4.9) compared to unqualified attendants (4.8). However, the prevalence of mistreatment is different depending on the on the type of provider. Unqualified attendants had higher rates of not informing women prior to a vaginal exam (*p* = 0.01) whereas qualified attendants were more likely to work in settings that did not allow birth companions (p = 0.01), and routinely performed enemas (0.001) and episiotomies (p = 0.001).

The Indian government recommends provision of maternity services by appropriately trained and qualified skilled birth attendants at health facilities. However, given the various context specific challenges in Uttar Pradesh, the prospect of all deliveries being cared for by qualified personnel at health facilities remains an important challenge. Therefore, it is important for policymakers to issue clear and comprehensive guidance on the role of unqualified providers at maternity facilities. Women that go to institutions for maternity care have a right to expect care from qualified personnel irrespective of whether it is the public or private sector. It is the duty of the government to protect those rights and design robust monitoring mechanisms to ensure that that unqualified personnel are not involved in provision of services.

Mistreatment was seen to coexist with limited adherence to evidence-based practices in this setting [27]. Saini et al. (2017) suggest that the primary drivers for poor care arise out of inequalities of information, wealth, and power [21]. In this context, we suggest that the drivers for mistreatment include resource-constraints, shortages of health workers, limited incentives, weak mentorship and supervision, restrictive institutional policies, lack of up-to-date knowledge, socio-economic factors and unequal power dynamics between health workers and pregnant women [45, 54–57]. Some researchers have also articulated that long-standing patterns of poor work-conditions, resource-scarcity, low-skills or overburdened health workers at facilities and limited choice for clients leads to poor QoC [16]. In addition, health workers may often not be aware of rights-based approaches or unable to provide high quality care despite their best intentions due to inherent organizational and work- environment related constraints, which are particularly relevant in this setting. Previous research has uncovered that there is a serious shortage of health workers in Uttar Pradesh. In fact, data from the National Sample survey (2011–2012) estimated that the density of doctors, nurses and midwives in Uttar Pradesh of 7.8 per 10,000 population was significantly below the WHO benchmark of 22.8 workers per 10,000 population [58].

While defining and measuring mistreatment, the concept of intentionality complicates measurement efforts. For example, some practices, such as fundal pressure or routine episiotomy are not evidence based and can be harmful [24, 50], but often health workers have been trained to do these things and think they are for the woman’s benefit. Are these indicators of mistreatment or of poor quality of care? Although, health workers may have been taught to use these interventions in the past, these harmful interventions are no longer recommended. Therefore, further conceptual clarity on the boundaries between mistreatment and poor quality of care is needed.

Another important finding of this study captured through observer’s comments was informal payments. Upon reflection, our QoC assessment tool should have specifically captured detailed information on informal payments. Informal payments can range from gratuity payments from appreciative patients, payments to jump the queue, receive better or additional care, to obtain drugs and commodities, or simply to receive any care at all [59]. Informal payments are considered to be inequitable and constitute institutionalised bribery, which may hamper the entire health system [59, 60]. Further, they tend to be prevalent in settings where health systems are under-funded, supervisory mechanisms are weak; where women are not empowered or not aware of their rights, and where providers are unlikely to face disciplinary action for their behaviours [59].

In summary, the literature suggests that mistreatment during labour and childbirth may be the result of many factors such as unfavourable institutional policies, resource and infrastructural constraints, socio-cultural factors, power differences between health workers and clients, limited knowledge and skills of health workers [6, 11, 56, 57]. We argue that non-adherence to clinical protocols such as through under treatment or overtreatment also constitute mistreatment of women at maternity facilities. An important question that emerges from our study is whether it is ethical to allow women to deliver in conditions where basic standards of evidence-based care, cleanliness, hygiene, dignity and equity are not met? The three districts where this study was conducted are not a part of the high priority districts of the Government of India. Therefore, it would be useful to conduct a similar study in high priority districts in Uttar Pradesh.

We demonstrated that mistreatment of women often occurs because of over-treatment and under-treatment which constitute a failures to adhere to professional standards of care [11]. Over-treatment and under-treatment should be considered in the global discourse on disrespect and abuse, as they are also a violation of human rights and constitute poor quality of care at maternity facilities. It is possible that some practices such as those related to individual health worker’s deficiencies in knowledge or skills are perhaps easier to change compared to long-standing socio-cultural factors that may give rise to mistreatment. Ultimately, mistreatment occurs, at least in part, because governments have not committed to or invested in participatory accountability mechanisms like social audits, community scorecards and others, which ensure that women’s experiences and perceptions of care are addressed and that respectful maternity care standards are followed [19]. This is one of the key recommendations emerging from this work.

### Limitations

This study used data from an observational study designed to capture descriptive information on elements of QoC for normal labour and childbirth. The study was not specifically powered to measure and explain mistreatment as a separate category of poor quality of care. Fieldworkers used open-ended comments to capture information that was contextually important or events that were particularly striking to them. Therefore, it is likely that the comments perhaps captured the more extreme events rather than routine care processes. There may also have been an observer bias, for example: comments recorded by observers perhaps reflects their own professional experiences, training and knowledge of respectful care practices. During fieldwork, we also noted that younger observers were more likely to take down detailed notes compared to the older observers, who were more experienced, and perhaps, more inclined to accept mistreatment as a normal occurrence. Our sample of private sector facilities was also limited by the fact that we had no official sampling frame for the private sector facilities in the study districts and that 13 private facilities refused to participate in the study. It is possible that the QoC of participating private sector facilities was different from other private facilities that were either not sampled or those that refused to participate. We have previously shown that any Hawthorne effect was negligible in this study since the aggregate quality scores for individual observers did not change depending on the order of observations [27]. Unfortunately, we do not have detailed information on pre or in-service trainings received by health workers at maternity facilities so we cannot draw firm conclusions on whether mistreatment arises due to individuals’ failure to change or due to inappropriate training opportunities. Although, we recognise that some indicators of mistreatment are of a much more serious than others, there were limitations in terms of assigning weights to these different indicators. While there are methods to assign intervention specific weights to different elements of quality of care such as Delphi techniques, consensus panels and nominal group processes, all of these methods have their own limitations including selection bias, poor validity and reliability.

The mixed methods approach taken to triangulate our findings, data collection round-the-clock on all seven days of the week, and the use of clinical practice observations are key strengths of this study.

## Conclusions

Mistreatment is common in both private and public sectors, albeit of different types. Efforts to expand institutional births in Uttar Pradesh and other high maternal and perinatal mortality settings would benefit from strengthening the quality of maternity care in both sectors so that evidence-based maternity care is provided, and positive births experiences are ensured. There are at least five specific recommendations emerging from this work. First, there needs to be a systematic and context-specific effort to measure mistreatment in high burden states in India in both public and private sectors. Second, a training initiative to orient all maternity care personnel to the principles of respectful maternity care would be useful. Third, systems to promote accountability for the application of respectful, woman-centred, maternity care pathways are needed. Fourth, participatory community and health system interventions need to be designed to articulate norms, standards of care and support the implementation of respectful maternity care standards. Lastly, we note that there needs to be a long-term, sustained investment in health systems so that supportive and enabling work-environments are available to front- line health workers.

## Abbreviations

ASHA: Accredited Social Health Activists
HMIS: Health Management Information systems
INR: Indian Rupees
JSY: Janani Suraksha Yojana
LMICs: Low- and Middle-income countries
LSHTM: London School of Hygiene and Tropical medicine
PHS: Public Healthcare Society
QoC: Quality of Care
UP: Uttar Pradesh
WHO: World Health Organization

## References

[1] Alkema L, Chou D, Hogan D, Zhang S, Moller AB, Gemmill A, Fat DM, Boerma T, Temmerman M, Mathers C *et al*: Global, regional, and national levels and trends in maternal mortality between 1990 And 2015, with scenario-based projections to 2030: a systematic analysis by the UN maternal mortality estimation inter-agency group. *Lancet (London, England)* 2016, 387(10017):462–474. 10.1016/S0140-6736(15)00838-7 PMC5515236 26584737

[2] Ng M, Misra A, Diwan V, Agnani M, Levin-Rector A, De Costa A (2014). An assessment of the impact of the JSY cash transfer program on maternal mortality reduction in Madhya Pradesh**,** India. Glob Health Action.

[3] Ministry of Health and Family Welfare: Janani SurakshaYojana: revised guidelines for implementation 2006. New Delhi: Government of India; 2006.

[4] Powell-Jackson T, Mazumdar S, Mills A (2015). Financial incentives in health: new evidence from India's Janani Suraksha Yojana. J Health Econ.

[5] Tuncalp Ö, Were WM, MacLennan C, Oladapo OT, Gulmezoglu AM, Bahl R, Daelmans B, Mathai M, Say L, Kristensen F (2015). Quality of care for pregnant women and newborns-the WHO vision. BJOG.

[6] Vogel JP, Bohren MA, Tuncalp O, Oladapo OT, Adanu RM, Balde MD, Maung TM, Fawole B, Adu-Bonsaffoh K, Dako-Gyeke P (2015). How women are treated during facility-based childbirth: development and validation of measurement tools in four countries - phase 1 formative research study protocol. Reprod Health.

[7] Campbell OM, Calvert C, Testa A, Strehlow M, Benova L, Keyes E, Donnay F, Macleod D, Gabrysch S, Rong L *et al*: The scale, scope, coverage, and capability of childbirth care. *Lancet (London, England)* 2016, 388(10056):2193–2208. 10.1016/S0140-6736(16)31528-8 27642023

[8] Vedam S, Stoll K, Rubashkin N, Martin K, Miller-Vedam Z, Hayes-Klein H, Jolicoeur G (2017). The mothers on respect (MOR) index: measuring quality, safety, and human rights in childbirth. SSM - Population Health.

[9] Declercq E, Sakala C, Corry M, Applebaum S, Herrlich A (2013). Listening to Mothers III: Report of the Third National US Survey of Women’s Childbearing Experiences.

[10] Baker SR, Choi PYL, Henshaw CA, Tree J (2005). 'I felt as though I'd been in jail': Women's experiences of maternity care during labour, delivery and the immediate postpartum. Fem Psychol.

[11] Bohren MA, Vogel JP, Hunter EC, Lutsiv O, Makh SK, Souza JP, Aguiar C, Saraiva Coneglian F, Diniz AL, Tuncalp O *et al*: The Mistreatment of Women during Childbirth in Health Facilities Globally: A Mixed-Methods Systematic Review. *PLoS Med* 2015, 12(6):e1001847; discussion e1001847. 10.1371/journal.pmed.1001847 PMC4488322 26126110

[12] Ishola F, Owolabi O, Filippi V (2017). Disrespect and abuse of women during childbirth in Nigeria: a systematic review. PLoS One.

[13] Warren C, Njuki R, Abuya T, Ndwiga C, Maingi G, Serwanga J, Mbehero F, Muteti L, Njeru A, Karanja J (2013). Study protocol for promoting respectful maternity care initiative to assess, measure and design interventions to reduce disrespect and abuse during childbirth in Kenya. BMC Pregnancy Childbirth.

[14] Sheferaw ED, Mengesha TZ, Wase SB (2016). Development of a tool to measure women's perception of respectful maternity care in public health facilities. BMC Pregnancy Childbirth.

[15] Chadwick RJ, Cooper D, Harries J (2014). Narratives of distress about birth in south African public maternity settings: a qualitative study. Midwifery.

[16] Bowser D, Hill K: Exploring evidence for disrespect and abuse in facility-based childbirth: report of a landscape analysis. In: *USAID-TRAction Project, Washington, DC.* edn.; 2010.

[17] D'Gregorio RP. Obstetric violence: a new legal term introduced in Venezuela. Elsevier. 2010. 10.1016/j.ijgo.2010.09.002 20926074

[18] Misago C, Kendall C, Freitas P, Haneda K, Silveira D, Onuki D, Mori T, Sadamori T, Umenai T (2001). From ‘culture of dehumanization of childbirth’to ‘childbirth as a transformative experience’: changes in five municipalities in north-East Brazil. Int J Gynecol Obstet.

[19] Freedman LP, Ramsey K, Abuya T, Bellows B, Ndwiga C, Warren CE, Kujawski S, Moyo W, Kruk ME, Mbaruku G (2014). Defining disrespect and abuse of women in childbirth: a research, policy and rights agenda. Bull World Health Organ.

[20] Chassin MR, Galvin RW (1998). The urgent need to improve health care quality. Institute of Medicine National Roundtable on health care quality. JAMA.

[21] Saini V, Brownlee S, Elshaug AG, Glasziou P, Heath I: Addressing overuse and underuse around the world. *Lancet (London, England)* 2017. 10.1016/S0140-6736(16)32573-9 28077230

[22] Oscarsson ME, Amer-Wahlin I, Rydhstroem H, Kallen K (2006). Outcome in obstetric care related to oxytocin use. A population-based study. Acta Obstet Gynecol Scand.

[23] Sharma G (2016). The changing paradigm of labour and childbirth in Indian cities: an enquiry into increasing rates of caesarean deliveries. Int J Epidemiol.

[24] Miller S, Abalos E, Chamillard M, Ciapponi A, Colaci D, Comande D, Diaz V, Geller S, Hanson C, Langer A (2016). Beyond too little, too late and too much, too soon: a pathway towards evidence-based, respectful maternity care worldwide. Lancet.

[25] White Ribbon Alliance: Respectful maternity care: the universal rights of childbearing women. Available at: http://whiteribbonallianceorg/wp-content/uploads/2013/10/Final_RMC_Charterpdf 2012, Accessed on 4.12.2016.

[26] Office of the Registrar General & Census Commissioner: Annual Health Survey 2012–2013 Fact Sheet, Uttar Pradesh**.** . In*.*; 2012–2013.

[27] Sharma G, Powell-Jackson T, Haldar K, Bradley J, Filippi V (2017). Quality of routine essential care during childbirth: clinical observations of uncomplicated births in Uttar Pradesh**,** India. Bull World Health Organ.

[28] Pomeroy AM, Koblinsky M, Alva S: Who gives birth in private facilities in Asia? A look at six countries. *Health Policy Plan* 2014, 29 Suppl 1(suppl 1):i38–i47. 10.1093/heapol/czt103 PMC4095919 25012797

[29] Thind A, Mohani A, Banerjee K, Hagigi F (2008). Where to deliver? Analysis of choice of delivery location from a national survey in India. BMC Public Health.

[30] Chaturvedi S, De Costa A, Raven J. Does the Janani Suraksha Yojana cash transfer programme to promote facility births in India ensure skilled birth attendance? A qualitative study of intrapartum care in Madhya Pradesh. Glob Health Action. 2015;8. 10.3402/gha.v8.27427 PMC4497976 26160769

[31] Chaturvedi S, Upadhyay S, De Costa A (2014). Competence of birth attendants at providing emergency obstetric care under India's JSY conditional cash transfer program for institutional delivery: an assessment using case vignettes in Madhya Pradesh province. BMC Pregnancy Childbirth.

[32] Coffey D (2014). Costs and consequences of a cash transfer for hospital births in a rural district of Uttar Pradesh, India. Soc Sci Med.

[33] Hulton LA, Matthews Z, Stones RW (2007). Applying a framework for assessing the quality of maternal health services in urban India. Soc Sci Med.

[34] Penn-Kekana L, Powell-Jackson T, Haemmerli M, Dutt V, Lange IL, Mahapatra A, Sharma G, Singh K, Singh S, Shukla V (2018). Process evaluation of a social franchising model to improve maternal health: evidence from a multi-methods study in Uttar Pradesh**,** India. Implement Sci.

[35] Nagarajan S, Paul VK, Yadav N, Gupta S (2015). The National Rural Health Mission in India: its impact on maternal, neonatal, and infant mortality. Semin Fetal Neonatal Med.

[36] Ministry of Health and Family Welfare: NHM Health management information system (HMIS) portal (https://mohfw.gov.in/sites/default/files/2858916361HMIS%20.pdf). 2015.

[37] Choices C (2005). In changing childbirth research N: routines in facility-based maternity care: evidence from the Arab world. BJOG.

[38] Getachew A, Ricca J, Cantor D, Rawlins B, Rosen H, Tekleberhan A, Bartlett L, Gibson H (2011). Quality of care for prevention and management of common maternal and newborn complications: a study of Ethiopia’s hospitals.

[39] World Health Organization: Integrated management of pregnancy and childbirth: World Health Organization.; 2003.

[40] International Institute for Population Sciences (IIPS) and Macro International: National Family Health Survey (NFHS-4), 2014–2015. 2014.

[41] Vyas S, Kumaranayake L (2006). Constructing socio-economic status indices: how to use principal components analysis. Health Policy Plan.

[42] Okafor II, Ugwu EO, Obi SN (2015). Disrespect and abuse during facility-based childbirth in a low-income country. Int J Gynaecol Obstet.

[43] Asefa A, Bekele D (2015). Status of respectful and non-abusive care during facility-based childbirth in a hospital and health centers in Addis Ababa**,** Ethiopia. Reprod Health.

[44] Sudhinaraset M, Treleaven E, Melo J, Singh K, Diamond-Smith N (2016). Women's status and experiences of mistreatment during childbirth in Uttar Pradesh: a mixed methods study using cultural health capital theory. BMC Pregnancy Childbirth.

[45] Kruk ME, Kujawski S, Mbaruku G, Ramsey K, Moyo W, Freedman LP. Disrespectful and abusive treatment during facility delivery in Tanzania: a facility and community survey. *Health Policy and Planning*. 2014:czu079. 10.1093/heapol/czu079 29304252

[46] Mitra A (2008). The status of women among the scheduled tribes in India. J Socio-Econ.

[47] Subramanian SV, Davey Smith G, Subramanyam M (2006). Indigenous health and socioeconomic status in India. PLoS Med.

[48] Chattopadhyay S (2018). The shifting axes of marginalities: the politics of identities shaping women's experiences during childbirth in Northeast India. Reprod Health Matters.

[49] Gupta J, Hofmeyr G, Smyth R (2007). Position in the second stage of labour for women without epidural anaesthesia (review).

[50] World Health Organization (1996). Care in Normal Birth: a practical guide.

[51] Hodnett ED, Gates S, Hofmeyr GJ, Sakala C (2013). Continuous support for women during childbirth. The Cochrane database of systematic reviews.

[52] Basevi V, Lavender T (2014). Routine perineal shaving on admission in labour. The Cochrane database of systematic reviews.

[53] Ministry of Health and Family Welfare: Skilled Birth Attendance-A Handbook for Auxiliary Nurse Midwives/Lady Health Visitors and Staff Nurses. 2010.

[54] Amoran O, Omokhodion F, Dairo M, Adebayo A (2004). Job satisfaction among primary health care workers in three selected local government areas in Southwest Nigeria. Niger J Med.

[55] Bosch-Capblanch X, Garner P (2008). Primary health care supervision in developing countries. Tropical Med Int Health.

[56] Jewkes R, Penn-Kekana L (2015). Mistreatment of women in childbirth: time for action on this important dimension of violence against women. PLoS Med.

[57] Sriram V, Topp SM, Schaaf M, Mishra A, Flores W, Rajasulochana SR, Scott K (2018). 10 best resources on power in health policy and systems in low- and middle-income countries. Health Policy Plan.

[58] Rao KD, Shahrawat R, Bhatnagar A (2016). Composition and distribution of the health workforce in India: estimates based on data from the National Sample Survey. WHO South East Asia J Public Health.

[59] Lewis M (2007). Informal payments and the financing of health care in developing and transition countries. Health Aff (Millwood).

[60] Gaal P, Belli PC, McKee M, Szocska M (2006). Informal payments for health care: definitions, distinctions, and dilemmas. J Health Polit Policy Law.

[61] Ambedkar BR. Castes in India: their mechanism, genesis and development. Readings in Indian Government And Politics Class, Caste, Gender. 2004:131–53.

